# Can Fall Risk Screening and Fall Prevention Advice in Hospital Settings Motivate Older Adult Patients to Take Action to Reduce Fall Risk?

**DOI:** 10.1177/07334648211004037

**Published:** 2021-04-02

**Authors:** Lotte M. Barmentloo, Vicki Erasmus, Branko F. Olij, Juanita A. Haagsma, Johan P. Mackenbach, Christian Oudshoorn, Stephanie C. E. Schuit, Nathalie van der Velde, Suzanne Polinder

**Affiliations:** 1 Erasmus MC, University Medical Center Rotterdam, the Netherlands; 2 Amsterdam UMC, University of Amsterdam, the Netherlands

**Keywords:** accidental falls, prevention and control, aged, hospitals, diagnosis

## Abstract

**Objective:**

We investigated whether an in-hospital intervention consisting of fall risk screening and tailored advice could prompt patients to take preventive action.

**Method:**

Patients (≥70) attending the emergency department and nephrology outpatient clinic in a Dutch hospital were screened. Patients at high risk received tailored advice based on their individual risk factors. Three months after screening, preventive steps taken by patients were surveyed.

**Results:**

Two hundred sixteen patients were screened. Of the 83 patients completing a 3-month follow-up, 51.8% took action; among patients who received tailored advice (*n* = 20), 70% took action. Patients most often adhered to advice on improving muscle strength and undergoing vision checkups (20%). Tailored advice and a reported low quality of life were associated with consulting a health care provider.

**Discussion:**

Patients at risk in these settings are inclined to take action after screening. However, they do not always adhere to the tailored prevention advice.

## Introduction

Falls and related injuries are major public health problems (Haagsma et al., 2016). For older adults, falls often lead to depleted daily life and social activities due to related injury and increased fear of falling (Gill et al., 2001; Tinetti & Williams, 1998). Thirty percent of adults aged ≥65 years experience a fall every year, and this rate increases with age (Rubenstein & Josephson, 2002; Turner et al., 2015). After a fall, 66% of older adults are injured, 20% to 30% visit a hospital, and 11% are admitted (Milat et al., 2011; Rubenstein & Josephson, 2002). Despite the growing attention to fall prevention and available fall prevention programs (Karlsson et al., 2013; Sherrington et al., 2017; Stubbs et al., 2015; Tricco et al., 2017), the number of fall-related emergency department (ED) visits and hospital admissions due to fall-related injuries keep rising (Burton et al., 2018; Cassell & Clapperton, 2013; Hartholt et al., 2010; Nilson et al., 2016; Shankar et al., 2017).

One challenge in prevention is the low adherence to related interventions among older adults (Merom et al., 2012). Barriers including fear of falling (Bunn et al., 2008), frailty (Malik et al., 2020), too time consuming (Child et al., 2012), no exercise history (Bunn et al., 2008), and transportation problems (Child et al., 2012; Malik et al., 2020) affect uptake and adherence to exercise programs. Furthermore, more general barriers such as a lack of awareness of existing programs (Bunn et al., 2008; Hill et al., 2014; Malik et al., 2020), patients’ perceptions of programs’ effectiveness (Bunn et al., 2008; Hill et al., 2014), denial of risk (Bunn et al., 2008), and underestimation of risk (Bunn et al., 2008; Hill et al., 2014) impede fall prevention implementation.

Self-perceived risk and awareness about risk status can positively affect implementation (Hill et al., 2014). Many older adults who have not yet experienced a fall are unaware of their increased risk of falling (Southerland et al., 2017; Vrolings & Gelissen, 2007). Therefore, identifying older adults with high fall risk is essential to create awareness among this population (Carpenter et al., 2014). Along with identifying risk, recognition of personal risk factors is important to offering tailored fall prevention advice. Advice tailored to the patient’s specific problems and needs increases its effectiveness (Ang et al., 2011; Bull et al., 1999) and adherence to fall prevention (Taylor et al., 2019).

Primary care providers such as physiotherapists and general practitioners (GPs) frequently provide care for older adults and, therefore, have great potential to detect risk and risk factors (Malik et al., 2020; Milisen et al., 2009). Apart from primary care providers, secondary care providers in hospital settings provide an opportunity to detect older adults with high fall risk (Carpenter et al., 2014; Close et al., 2012; Huded et al., 2015). However, contrary to international guidelines (Carpenter et al., 2014; Centre for Clinical Practice at Nice, 2013; Joint Commission International, 2017; Weigand & Gerson, 2001), in-hospital fall prevention is not yet standardized in the Netherlands. Furthermore, studies on the implementation of such guidelines in outpatient settings are lacking. At present, older adults are more frequently screened only at EDs for fall risk (Carpenter et al., 2014). Meanwhile, patients with chronic diseases not directly resulting from a fall could also be at a potentially high risk of falling (Lawlor et al., 2003). Such patients tend to have more contact within the hospital than patients visiting the ED and, therefore, build a stronger relationship with their specialist, affecting the uptake of and adherence to advice (Menting et al., 2019).

Because guidelines are not well implemented, the positive effect of self-perceived risk, awareness, and tailored advice in hospital settings remains inadequately studied. To map risk and risk factors of this specific cohort and provide patients with tailored advice, this study sought (a) to explore fall risk and risk factors of patients in two hospital settings (i.e., ED and outpatient clinic). We investigated (b) whether a hospital-based fall risk assessment followed by tailored prevention advice can prompt patients to take action to reduce their fall risk and (c) which patient characteristics are associated with taking action after screening. We performed this screening at an ED and a nephrology outpatient clinic (NOC) of a university teaching hospital to assess both patients in general and patients with chronic diseases.

## Method

### Study Design and Population

This observational cohort study was performed from December 2016 to June 2017. Interested patients were recruited in the waiting room within the first 3 months. Following existing Dutch guidelines on screening older adults in a hospital, patients aged ≥70 years who visited the NOC or ED of the Erasmus Medical Center in Rotterdam, the Netherlands, were invited for a fall risk screening. The null hypothesis was to find no relationship between guideline adherence and determinants of guideline adherence. For the calculation of the sample size, we set the threshold probability for rejecting the null hypothesis (α two-tailed) at .05. To prove guideline adherence, it was required that 38 patients at high risk of falls participate. Considering an average high fall risk of 37.5%, nonresponse (estimated at 65%), and dropouts in fall prevention (estimated at 15%), at least 183 patients had to be included. Patients were screened in these departments because of the larger number of frail older patients visiting and the relevant comorbidity pertaining to falls. Exclusion criteria were (a) not understanding the Dutch language and (b) incapacitation. Patients screened at the NOC were informed about the study by one of the researchers; those interested provided informed consent immediately after screening. Patients screened at the ED received information about the study and provided informed consent by mail. In addition to data collection in the departments, data were also gathered by a survey 2 weeks and 3 months after screening. The medical ethics committee of Erasmus MC, University Medical Center Rotterdam, provided ethical approval (number 2016-666).

The study included intervention and data collection by survey. In the intervention, patients were screened for fall risk at two hospital departments. Patients with low fall risk received a flyer clarifying that, at the moment of screening, they did not have high fall risk. Patients with high fall risk were contacted for a comprehensive fall risk analysis to identify the risk factors present. They received personal fall prevention advice based on their risk factors. For data collection by survey, the patients screened received two surveys regarding patient characteristics and the actions patients took to prevent falling.

### Intervention

#### Fall risk screening

In both departments, the Dutch fall risk test was used to screen older adults for fall risk. It is based on three factors mentioned in the existing literature, which are most frequently associated with recurrent falls, namely, (a) a history of falls and (b) problems with movement and balance (Stalenhoef et al., 2002). The fall risk test comprises three questions: (a) Did you fall during the past 12 months? (b) Do you experience problems with movement and balance? and (c) Are you afraid of falling? The first question can be answered with no, yes, once, or yes, multiple times, and the other questions with no or yes. The fall risk test labeled a patient answering “yes” to the first question or to two of the three questions as high fall risk (National Guidelines VeiligheidNL, n.d.). Within the Netherlands, this test is recommended to screen community-dwelling older adults (VeiligheidNL, 2017).

#### Fall risk analysis to inform individual prevention advice

For patients with high fall risk, screening also involved a comprehensive fall risk analysis by telephone. This analysis was performed within 2 weeks after the initial screening by a trained research nurse and aimed to identify personal risk factors associated with high fall risk to compare risk factors between departments and target further preventive activities. The analysis comprised questions on 12 known fall risk domains that were determined based on existing questionnaires (Katz & Akpom, 1976; VeiligheidNL, 2015) and expert opinion. The risk domains used were prescription drug use (cardiovascular medication and psychotropic medication), poor mobility and balance, fall history, painful feet, poor vision, fall hazard in one’s own living environment, painful joints, fear of falling, osteoporosis, dizziness, challenges performing daily living activities, and poor memory and concentration. An overview of the risk domains and when a domain was considered a risk factor are in the supplemental material.

#### Tailored prevention advice

After screening, patients with low fall risk received a flyer informing them they had low fall risk and could consult a GP for further questions. Patients undergoing the comprehensive analysis received tailored prevention advice by post based on risk factors. For the medication, fall history, painful feet, osteoporosis, and challenges performing daily living activities, patients were advised to meet a GP. The domains poor mobility and balance, poor vision, fall hazards in one’s own living environment, painful joints, fear of falling, dizziness, and poor memory and concentration each carried specific advice. For example, when “mobility and balance” was a risk factor, patients received advice on two multifactorial fall prevention programs located near their homes. An overview of risk domains, definitions of risk factors, advice, and type of action linked to the advice are in the supplemental material.

### Data Collection

Follow-up data by survey were collected at two time points: 2 weeks post–initial screening at the ED and NOC and at 3 months of follow-up. Two weeks after initial screening, all patients received a survey by post or email. For patients with high fall risk, this survey was sent after a comprehensive analysis. This survey included sociodemographic questions on age, sex, ethnicity, whether living independently or with partner or children, and education level. Patients were considered Dutch when born in the Netherlands, and immigrant if the patient or one parent was born elsewhere. Education level was categorized as low (below primary school, primary school, or little more than primary school), intermediate (i.e., technical school, vocational education, general secondary/preuniversity education), and high (i.e., college/university). They were asked about chronic conditions, and eight options were listed to which they answered yes or no. An open question was included to note other chronic conditions. The total chronic conditions were thus calculated. Health-related quality of life was assessed by the five-dimensional EuroQol instrument (EQ-5D-5L + cognition; Hoeymans et al., 2005), in which a utility score was calculated using the Dutch tariff, with scores ranging from 0 (death) to 1 (full health; Versteegh et al., 2016). In addition, patients could rate their own health on the Visual Analog Scale (VAS [0–100]) for quality of life.

At 3 months follow-up, a second questionnaire was sent to assess the preventive actions undertaken, asking the following: (1) Did you undertake any fall prevention action without help of a health care professional? (2) Did you consult a GP about fall prevention? and (3) Did you consult a medical specialist regarding fall prevention? Patients could indicate whether they had undertaken any of these fall preventive actions with the following answers: looked up or received information, performed mobility training to improve muscle strength and/or endurance, performed mobility training to improve skills, had eyes tested, made changes to shoes, made adjustments in and around the house, received lifestyle advice, and stopped or changed medication. To determine adherence, these actions were compared between departments and with the postscreening advice.

### Statistical Analyses

Baseline and follow-up characteristics were expressed as mean and standard deviation for continuous variables and as numbers and percentages for dichotomous variables. Differences in baseline characteristics and preventive actions between participants with low and high risk were compared using a Mann–Whitney U test for continuous variables and a chi-square test for dichotomous variables. The chi-square was also used for comparison between departments on fall risk factors and the actions patients undertook. Adherence to advice was expressed as percentages. To investigate which characteristics, regardless of risk, were associated with taking action, logistic regression analyses were used. A univariate model was used to determine the relationship between characteristics and undertaking action. We could not collect a clear set of characteristics from the literature, which could be expected to be associated with taking action. Therefore, we included as independent variables all baseline characteristics and whether patients received tailored advice, after which all characteristics with a significance level of <.20 were selected for a multivariable model. Variables were included in the multivariate model using the Enter method. Taking action, taking action with a health care worker, and taking action independently were used as dependent variables; the variable “help from a health care worker” merged help from a GP and a specialist. For assessing model goodness-of-fit, Nagelkerke *R*^2^ was used. The discriminative ability of the models is quantified with the area under the curve (AUC); *p* < .05 was considered statistically significant. Analyses were performed using SPSS Statistical Data software (IBM) version 25.

## Results

**Figure 1.**
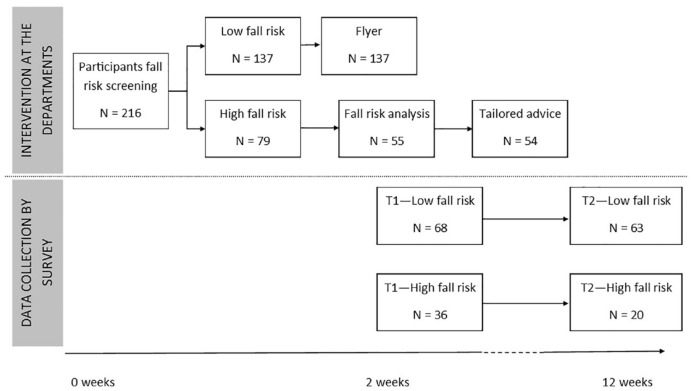
Patient flowchart.

Fall risk screening was performed for 216 patients, most of whom were patients from the ED (*n* = 116; Figure 1). Seventy-nine participants (36.6%) had high fall risk, 77 (35.6%) indicated they had experienced a fall in the previous year, and 34 (15.7%) participants had fallen twice or more. Whereas 112 participants (51.9%) had mobility problems, 58 (26.9%) indicated fear of falling. No difference in risk was seen between patients attending the ED and NOC; however, the frequency of falls in the last 12 months was higher among patients attending the ED (ED: 42.2% vs. NOC: 28.3%, *p* = .033).

### Characteristics and Health-Related Quality of Life

Of 216 patients screened, 104 (48.1%) responded to the baseline survey (T1). Of these, 68 (65.4%) had low fall risk and 36 (34.6%; *p* = .002) a high risk. Baseline characteristics of these patients, collected at T1, are in Table 1. Patients with high risk had more problems in all domains regarding reported health-related quality of life, with the largest difference being for mobility (high: 97.1% vs. low: 47.7%, *p* ≤ .001). Participants with high fall risk had a significantly lower EQ-5D utility score than participants with low fall risk (high: 0.50 vs. low: 0.80, *p* ≤ .001). In addition, patients with high risk had lower VAS scores (high: 55 vs. low: 70, *p* ≤ .001). Furthermore, a difference in the prevalence of chronic conditions was observed. Patients with high fall risk suffered more often from two (high: 31.4% vs. low: 14.1%, *p* = .040) and three or more (high: 54.3% vs. low: 12.5%, *p* ≤ .001) chronic conditions compared with patients with low fall risk.

**Table 1.** Baseline Characteristics With Differences Between Patients With High and Low Fall Risk.

Characteristics *N* (%)	Total *N* = 104	Low-risk patients *N* = 68 (65%)	High-risk patients *N* = 36 (35%)	Difference, *p*^a^
Department				.633
ED	29 (27.9%)	20 (29.4%)	9 (25%)	
NOC	75 (72.1%)	48 (70.6%)	27 (75%)	
Sex (male)^b^	74 (72.5%)	52 (77.6%)	22 (62.9%)	.113
Dutch nationality (yes)^c^	88 (89.8%)	59 (89.4%)	29 (90.6%)	.850
Living together with partner or children (yes)^c^	75 (76.5%)	52 (81.3%)	23 (67.6%)	.130
Education^d^				
Low	49 (52.1%)	35 (54.7%)	14 (46.7%)	.468
Intermediate	27 (28.7%)	17 (26.6%)	10 (33.3%)	.499
High	18 (19.1%)	12 (18.8%)	6 (20%)	.886
Living situation^e^				
Independent	84 (86.6%)	58 (92.1%)	26 (76.5%)	.031
Independent + care	11 (11.3%)	4 (6.3%)	7 (20.6%)	.035
Care institution	2 (2.1%)	1 (1.6%)	1 (2.9%)	.654
Chronic conditions^f^				
0	18 (18.2%)	17 (26.6%)	1 (2.9%)	.003
1	32 (32.3%)	28 (43.8%)	4 (11.4%)	.001
2	20 (20.2%)	9 (14.1%)	11 (31.4%)	.040
3 or more	27 (27.3%)	8 (12.5%)	19 (54.3%)	<.001
EQ-5D + cognition^g,h^				
Problems mobility	65 (65%)	31 (47.7%)	34 (97.1%)	<.001
Problems self-care	23 (23%)	5 (7.7%)	18 (51.4%)	<.001
Problems daily activities	47 (47%)	21 (32.3%)	26 (74.3%)	<.001
Pain/discomfort	68 (68%)	39 (60%)	29 (82.9%)	.019
Anxiety/depression	33 (33%)	17 (26.6%)	16 (44.4%)	.068
Cognition^i^	48 (47.5%)	27 (41.5%)	21 (58.3%)	.105
*M* (*SD*)				
Age	75.0 (4.6)	74.4 (4.2)	75.9 (5.3)	.189
EQ-5D-5L utility^c^	0.69 (0.30)	0.80 (0.23)	0.50 (0.33)	<.001
VAS^j^	65 (19.4)	70 (17.8)	55 (18.8)	<.001

*Note.* ED = emergency department; NOC = nephrology outpatient clinic; EQ-5D-5L = five-dimensional EuroQol instrument + cognition; VAS = Visual Analog Scale.

a A Mann–Whitney *U* test was used for continuous data and a chi-square test for categorical data. ^b^*N* =102. ^c^*N* = 98. ^d^*N* = 94. ^e^*N* = 97. ^f^*N* = 99. ^g^For the EQ-5D, domains were listed as a problem when patients answered they had slight problems regarding the domain or more than slight problems. ^h^*N* = 100. ^i^*N* = 101. ^j^*N* = 99.

*p* < .05 is considered statistically significant.

### Fall Risk Factors

Of all patients with high fall risk (*n* = 79), 55 (69.6%) participated in the comprehensive analysis that identified personal risk factors. Most of these 55 high-risk patients were at risk in the mobility and balance domains (92.7%) and medication use (92.5%). Furthermore, a history of falls was a common risk factor (85.5%). Table 2 presents an overview of all risk factors. The risk factors medication and painful feet were present more often in patients from the NOC compared with patients from the ED, whereas dizziness was less frequent in patients from the NOC vis-à-vis patients from the ED.

**Table 2.** Fall Risk Factors Present in Patient With High Risk From the ED and the NOC and Difference Between Patients From the Two Departments.

Fall risk factor	Total, *N* = 55 *N* (%)	ED, *N* = 23 *N* (%)	NOC, *N* = 32 *N* (%)	Chi-square *p*
Mobility and balance	51 (92.7%)	21 (91.3%)	30 (93.8%)	.730
Medication^a^	49 (92.5%)	17 (81%)	32 (100%)	.010
Fall history	47 (85.5%)	21 (91.3%)	26 (81.3%)	.297
Vision^a^	31 (57.4%)	13 (59.1%)	18 (56.3%)	.836
Dizziness	30 (54.5%)	17 (73.9%)	13 (40.6%)	.014
Painful joints	30 (54.5%)	13 (56.5%)	17 (53.1%)	.803
Painful feet	29 (52.7%)	8 (34.8%)	21 (65.6%)	.024
Living environment	29 (52.7%)	11 (47.8%)	18 (56.3%)	.537
Fear of falling	27 (49.1%)	11 (47.8%)	16 (50%)	.874
ADL	22 (40%)	9 (39.1%)	13 (40.6%)	.911
Osteoporosis	19 (34.5%)	5 (21.7%)	14 (43.8%)	.090
Cognition	18 (32.7%)	10 (43.5%)	8 (25%)	.150

*Note.* A chi-square test was used for comparing the presence of risk factors in patients from the ED and the nephrology department. ED = emergency department; NOC = nephrology outpatient clinic; ADL = challenges performing daily living activities.

a Number of risk factors present according to the additional fall analysis, administered to 55 of the 79 patients who were considered at high risk of falls.

*p* < .05 is considered statistically significant.

### Preventive Actions

**Figure 2.**
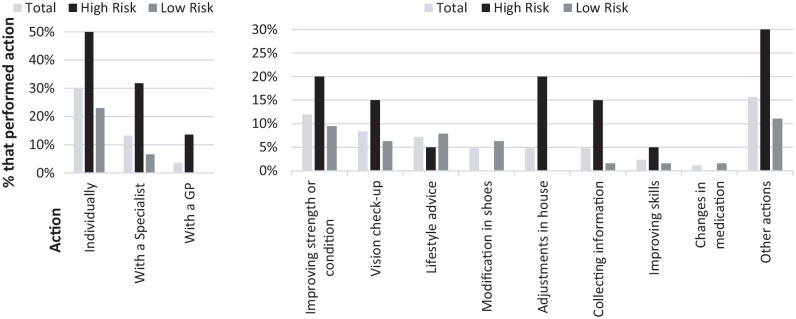
Percentage of patients indicating they had taken action after a hospital fall risk screening. *Note.* GP = general practitioner.

In all, 83 participants (low-risk *n* = 63 and high-risk *n* = 20) responded to the 3-month follow-up, with 51.8% indicating they had undertaken action to prevent falling following the screening. Of patients who had low fall risk and thus did not receive fall prevention advice, 46% indicated doing something to prevent falls, which was fewer than in the group receiving tailored advice (70%). Of the abovementioned 83 patients, 25 (30.1%) performed a preventive action without help from a health care provider, 11 (13.3%) contacted a medical specialist, and three (3.6%) contacted their GP for fall prevention. Figure 2 shows an overview of actions performed by patients after screening. Strength and endurance training was undertaken most often (12.0%), with vision checkups (8.4%) being next in frequency. Of the 20 patients with high risk who received personal prevention advice, strength and endurance training together with adjustments in and around the house ranked first in frequency (20%); vision checkup and information collection (15%) stood second.

The actions patients undertook did not always align with the tailored advice they received. When patients were advised to consult a GP, 16.7% did so, whereas 42.1% visited another health care provider to prevent falling. When specific advice was given, patients adhered most often to improving strength and or balance (22.2%), but less to vision checkup (20.0%), training to improve skills (12.5%), and adjustments in and around the house (10%).

### Which Patients Take Action

Increasing age, presence of chronic conditions, tailored prevention advice, and a reported poorer quality of life are associated with taking action to prevent falls (Table 3). However, after controlling for other characteristics in a multivariate model (Nagelkerke *R*^2^ = .313, AUC = .792), the effect of the tailored advice disappears and only the presence of chronic conditions is associated with a higher likelihood of taking action after screening (odds ratio [OR] = 7.37, 95% confidence interval [CI] = [1.32, 41.06], *p* = .023). Both tailored advice (OR = 10.14, 95% CI = [2.12, 48.42], *p* = .004) and lower EQ-5D utility score (OR = 0.070, 95% CI = [0.01, 0.91], *p* = .042) are associated with taking action with a health care provider’s aid, after controlling for other characteristics in a multivariate model (Nagelkerke *R*^2^ = .391, AUC = .857).

**Table 3.** Association Between Preventive Action and Participant Characteristics.

Characteristics	Univariate model			Multivariate model			AUC
	Pseudo *R*^2^	OR [95% CI]	*p*	Pseudo *R*^2^	OR [95% CI]	*p*	
Action: Did something to prevent falling							
Age	.093	1.133 [1.019, 1.260]	.021		1.134 [0.997, 1.291]	.056	
EQ-5D utility score	.159	0.044 [0.004, 0.428]	.007		0.260 [0.022, 3.113]	.260	
Chronic conditions	.175	9.250 [1.923, 44.503]	.006		7.373 [1.324, 41.055]	.023	
Tailored advice yes	.056	2.833 [0.975, 8.231]	.067	.313	1.228 [0.336, 4.490]	.765	.792
Action: Went to health care worker to prevent falling							
Female	.036	2.366 [0.660, 8.481]	.186		1.087 [0.199, 5.935]	.923	
EQ-5D utility score	.117	0.038 [0.005, 0.322]	.003		0.070 [0.005, 0.913]	.042	
Tailored advice yes	.142	10.545 [2.700, 41.184]	.001	.391	10.140 [2.123, 48.421]	.004	.857
Action: Undertake actions to prevent falling without a health care worker							
Dutch nationality	.048	0.304 [0.074, 1.254]	.100		0.227 [0.036, 1.427]	.114	
EQ-5D utility score	.066	0.178 [0.030, 1.064]	.058		0.427 [0.046, 3.933]	.452	
Chronic conditions	.105	7.700 [0.948, 62.528]	.056		5.930 [0.552, 63.710]	.142	
Tailored advice yes	.090	3.556 [1.218, 10.376]	.020	.227	2.849 [0.797, 10.181]	.107	.725

*Note.* Nagelkerke *R*^2^ is used for assessing model goodness-of-fit. AUC is used to quantify discriminative ability of the models. AUC = area under the curve; OR = odds ratio; CI = confidence interval; EQ-5D = five-dimensional EuroQol instrument.

## Discussion

The fall risk screening at both ED and NOC revealed an equally large percentage of older adults with high fall risk. These patients with high risk had a poorer reported quality of life and substantially more mobility problems and more of them suffered from comorbidity than patients with low risk. Most fall risk problems were in the mobility and balance, medication, and fall history domains. After screening, more than half the patients took action to prevent falls but not always according to the tailored advice. When patients adhered to the advice, it was most often to improve balance or strength or have their eyes tested. Although tailored advice was not associated with undertaking fall prevention actions in general, it was associated with consulting health care providers about fall prevention.

When patients were at risk of falling and received individual prevention advice, 70% took action to prevent falling. Considering the relatively simple intervention, these percentages hold out hope. Elliott et al. (2012) found similar percentages (73%). However, Elliot’s participants visited a fall prevention event of their own accord, suggesting prior motivation regarding fall prevention. A study in an ED setting by Phelan et al. (2016) also found slightly higher percentages (73%–79%) of patients undertaking preventive action. However, these patients were included after a fall. This could have increased perceived personal relevance and motivation to change. Shah et al. (2006) found much lower percentages after screening and educational information (15%). In the study of Shah et al., educational information was generalized instead of tailored, thus potentially affecting uptake (Taylor et al., 2019). In the current study, tailored advice was not always adhered to. However, a slight increase in intensity, such as information, physical tests, and one-on-one reviews of personal recommendations prompted considerably higher adherence percentages (Baker et al., 2019), as did a few home visits (Taylor et al., 2019).

Patients with high risk who received tailored advice more often undertook preventive action. However, after correcting for other characteristics, the presence of chronic conditions was associated with such action rather than tailored advice. That chronic conditions were associated with undertaking action can be seen as remarkable, because a medical condition normally is associated with limited physical activity among older adults (Murphy et al., 2002; Picorelli et al., 2014). Contrarily, patients with chronic conditions are already more aware of their health status and in contact with health care professionals and, perhaps, are more inclined to undertake action, whereas healthy older adults might not identify with fall prevention and, therefore, do not undertake action. High fall risk and thus tailored advice are predictors for consulting a health care provider for fall prevention and, after receiving advice to visit a GP, 42% did visit a health care provider. Regrettably, because the advice was given for multiple risk factors, we do not know whether patients consulted with the health care provider for the specific risk factor we advised.

Screening on fall risk is much more common in ED settings than at other hospital departments (Carpenter et al., 2014). To the researchers’ knowledge, this is the first study to investigate fall risk and actions of patients in two different hospital settings. Given the finding that no differences in fall risk were seen between patients screened in ED or NOC, fall risk among older adult patients should receive the same degree of attention within an outpatient department. Furthermore, the study did not focus on those on a fall-related visit, but rather on primary prevention by inviting all older adults visiting either of the two departments.

This study has several limitations. Although many patients took fall prevention action after screening, we do not know what exactly prompted such action. It could be not only the screening itself but also the additional tailored prevention advice that inspired patients. Furthermore, it is unknown whether this was the first time the patients were confronted with their fall risk status. Because we do not know whether the information regarding their risk status was new to the patient, it is hard to say at follow-up whether this has been the (only) trigger for action. Moreover, we were unable to perform a pre- versus postscreening comparison because comparable data of fall risk and preventive actions were not assessed at baseline. Another limitation is the low response rate to the second questionnaire, which made the follow-up cohort, particularly of patients at high risk, quite small. Although based on our sample size calculation, an adequate number of older adults were invited (216 instead of 183), due to dropouts, we were eventually short of participants. The assessed fall risk among this population (37.5%), response rate (65%), and dropouts expected until baseline (15%) were all estimated well. However, among the high-risk population, the dropout rate from baseline until follow-up was higher than expected (at 42% instead of 15%). Patients dropping out were those with chronic conditions and a poorer health-related quality of life—aspects that are associated with performing preventive actions or preventive actions with help from a health care worker; so, this might have affected outcomes. The high dropout rates also hinder drawing conclusions about patients’ adherence to the specific advice and whether some advice was adhered to better than others. We noticed that, within hospital settings, compliance among older adults is low and retaining the cohort seems difficult. Finally, there was an unequal distribution of participants from the two departments. Patients attending NOC appeared more inclined to participate. This could be due to the process of obtaining informed consent, which was done immediately after screening at the NOC but by post at the ED. Besides, patients attending ED were present because of an acute situation concerning their health, which may have affected their willingness to participate.

For future in-hospital fall prevention programs, it is important to retain the cohort. To do so, additional analyses should be scheduled directly after screening. Studies could investigate adherence to specific advice, using a larger cohort. Furthermore, building on the risk factors presented in the current study, future studies could investigate whether these factors can predict future falls. However, adherence to fall prevention remains a major challenge in which health care providers are key. A bit more personal attention could potentially increase adherence to given advice.

## Conclusion

Within this hospital population, a large percentage had high fall risk. This indicates that besides ED, departments with patients with chronic disease also have great potential to screen older adults for fall risk. Patients who receive tailored advice are motivated to undertake action to prevent falling. In particular, patients with high risk who received tailored advice are more likely to consult a health care provider. With more personal attention from health care providers, interventions have the potential to also increase adherence. However, future research should investigate why patients do or do not adhere to such tailored advice.

## Supplemental Material

sj-pdf-1-jag-10.1177_07334648211004037 – Supplemental material for Can Fall Risk Screening and Fall Prevention Advice in Hospital Settings Motivate Older Adult Patients to Take Action to Reduce Fall Risk? Click here for additional data file. Supplemental material, sj-pdf-1-jag-10.1177_07334648211004037 for Can Fall Risk Screening and Fall Prevention Advice in Hospital Settings Motivate Older Adult Patients to Take Action to Reduce Fall Risk? by Lotte M. Barmentloo, Vicki Erasmus, Branko F. Olij, Juanita A. Haagsma, Johan P. Mackenbach, Christian Oudshoorn, Stephanie C. E. Schuit, Nathalie van der Velde and Suzanne Polinder in Journal of Applied Gerontology
