# Supplementary material for: Can Fall Risk Screening and Fall Prevention Advice in Hospital Settings Motivate Older Adult Patients to Take Action to Reduce Fall Risk?
Source: J Appl Gerontol. 2021 Apr 2;40(11):1492–501. doi: 10.1177/07334648211004037 (PMC8564248; doi:10.1177/07334648211004037)
Supplement: sj-pdf-1-jag-10.1177_07334648211004037 – Supplemental material for Can Fall Risk Screening and Fall Prevention Advice in Hospital Settings Motivate Older Adult Patients to Take Action to Reduce Fall Risk? [file sj-pdf-1-jag-10.1177_07334648211004037.pdf]

Supplemental material*:* Fall risk factors, advice and action

Risk domain	When a risk factor	Advice	Action
**Medication**	Psychotropic or cardiovascular medication	General practitioner	Visit GP
**Mobility and balance**	Problems with movement, balance or the use of walking aids.	Fall prevention programs in balance or falls in the past fall with application form.	Program to improve strength and/ or condition
**Feet**	Painful feet	General practitioner	Visit GP
**Vision**	Difficulty reading or looking far away or last year never let the eyes checked	Tips: have you eyes measured every year and keep your glasses close while resting or sleeping.	Eyes measurement
**Living environment**	>1 answered with no<br>• Use of solid stepladder instead of chairs<br>• Sturdy footwear<br>• Doorsteps removed or chamfered<br>• House properly lit<br>• Lights on during the night<br>• Loose rugs with anti-slip<br>• Raised chairs, bed and sofa's.<br>• Anti-slip in bathroom and toilet<br>• Support in bathroom and toilet<br>• Handrails on both sides of the stairs	A form with adjustments<br>• The use of a stepladder instead of chair or stool<br>• Remove or skew doorsteps<br>• Turn on the light when leaving your bed during nights<br>• Remove loose rugs or attach anti-slip<br>• A good chair or sofa helps with getting up, is raised and with armrest. Chose a high bed with support.<br>• Take care of good support<br>• It is most save with handrails on both sides of the stairs	Adjustments in the house
**Joints**	Painful joints	• Regular exercise<br>• Alternate day with rest and activities<br>• Walking aids<br>• Talk about possible painkillers with your GP	Visit GP
**Fear of falling**	Worried, insecure or anxious to fall	Fall prevention program a matter of balance with application form	Program to improve skills
**Osteoporosis**	>2 points<br>• BMI <20 = 1<br>• 1 bone fracture for fiftieth = 1<br>2 bone fractures for fiftieth = 2<br>• Bone fracture within last 2 years = 4<br>• Swirl collapse = 4	General practitioner	Visit GP

	• Fallen > 2 times last year = 1<br>• Broken hip with one of your parents = 1<br>• Prednisone for more than 3 months = 4<br>• The use of vitamin D = 1		
**Dizziness**	Suffer from dizziness (vertigo)	• Try to keep doing daily things<br>• Do not suddenly get up out of bed or chars, first contract muscles<br>• Maintain muscle strength and condition by regular walking or cycling<br>• Do not wear reading glasses while walking around<br>• If you suddenly feel dizzy, it is best to sit down<br>• Eat healthy, to maintain glucose levels<br>• Do not smoke and drink little/no alcohol	No specific action
**Memory**	Memory complaints	• If you want to concentrate, make sure you're not getting distracted<br>• Do one thing at the same time<br>• Try to cycle or walk for half an hour each day	No specific action
**Challenges performing daily life activities**	Not able to wash, dress, move, visit the toilet or eat alone or is incontinent for urine	General practitioner	Visit GP
**History of falls**	Fallen in the last 12 months	General practitioner	Visit GP

Fall risk domains with explanation when the risk domain counts as risk factor (when a risk factor), which advice is given (advice) and which action is linked to the advice (action).
